# Supplementary material for: Programming viscoelastic properties in a complexation gel composite by utilizing entropy-driven topologically frustrated dynamical state
Source: Nat Commun. 2024 Apr 26;15:3569. doi: 10.1038/s41467-024-47969-z (PMC11053056; doi:10.1038/s41467-024-47969-z)
Supplement: Supplementary file 1 — Supplementary Information [file 41467_2024_47969_MOESM1_ESM.pdf]

Supplementary Information for

Programming viscoelastic properties in a complexation gel composite by utilizing entropy-driven topologically frustrated dynamical state

Gui Kang Wang[1,2], Yi Ming Yang[1,2], Di Jia[1,2 *]

1. Beijing National Laboratory for Molecular Sciences, Laboratory of Polymer Physics and Chemistry, Institute of Chemistry Chinese Academy of Sciences, Beijing 100190, China;

2. University of Chinese Academy of Sciences, Beijing 100049, China

*Corresponding author: Di Jia: jiadi11@iccas.ac.cn

Experimental Section

Materials. Sodium acrylate, Acrylamide (40% w/v), Tetramethyl-ethylenediamine (TEMED), Ammonium persulfate (APS) and Poly(diallyldimethylammonium chloride) (PDA) of three molecular weights (400-500kDa, 200-350kDa, and <100kDa) were bought from Sigma-Aldrich and used as received. Bis-Acrylamide (2% w/v) was purchased from Macklin. All materials were used without further purification. Hydrophilic Polyvinylidene Fluoride (PVDF) filters with pore size 220nm were purchased from Millex Company. Deionized water was obtained from a Milli-Q water purification system (Merk Millipore IQ 7000). The resistivity of deionized water used was 18.2 MΩ·cm.

Swelling ratio measurement. The as-prepared gel composites were synthesized in the Petri dish so that the sample volume can be precisely measured through the diameter and thickness of gel composites by a vernier scale. The as-prepared gel composites were immersed in either deionized water or PDA solutions, whose PDA concentration is the same as that inside the gel composites. The samples were swelled for more than one week until their volumes no longer changed anymore, indicating they reached swelling equilibrium.

Rheology. Shear storage (G') and loss (G'') moduli were obtained using a stress-controlled rheometer (Anton parr MCR 502), using a 25 mm roughened parallel plate geometry. A frequency sweep from 100 to 0.1 rad/s was performed with a shear strain of γ=1%, which is in the linear viscoelastic region.

Preparation of dust-free DLS tubes. Since dynamic light scattering (DLS) measurement is extremely sensitive to dust, the DLS tubes were first washed several times with pure water and acetone separately. After they were dried in the oven overnight, aluminum foil was used to wrap up the tubes and then these tubes were further cleaned by distilled acetone through an acetone fountain setup.

Supplementary Figures

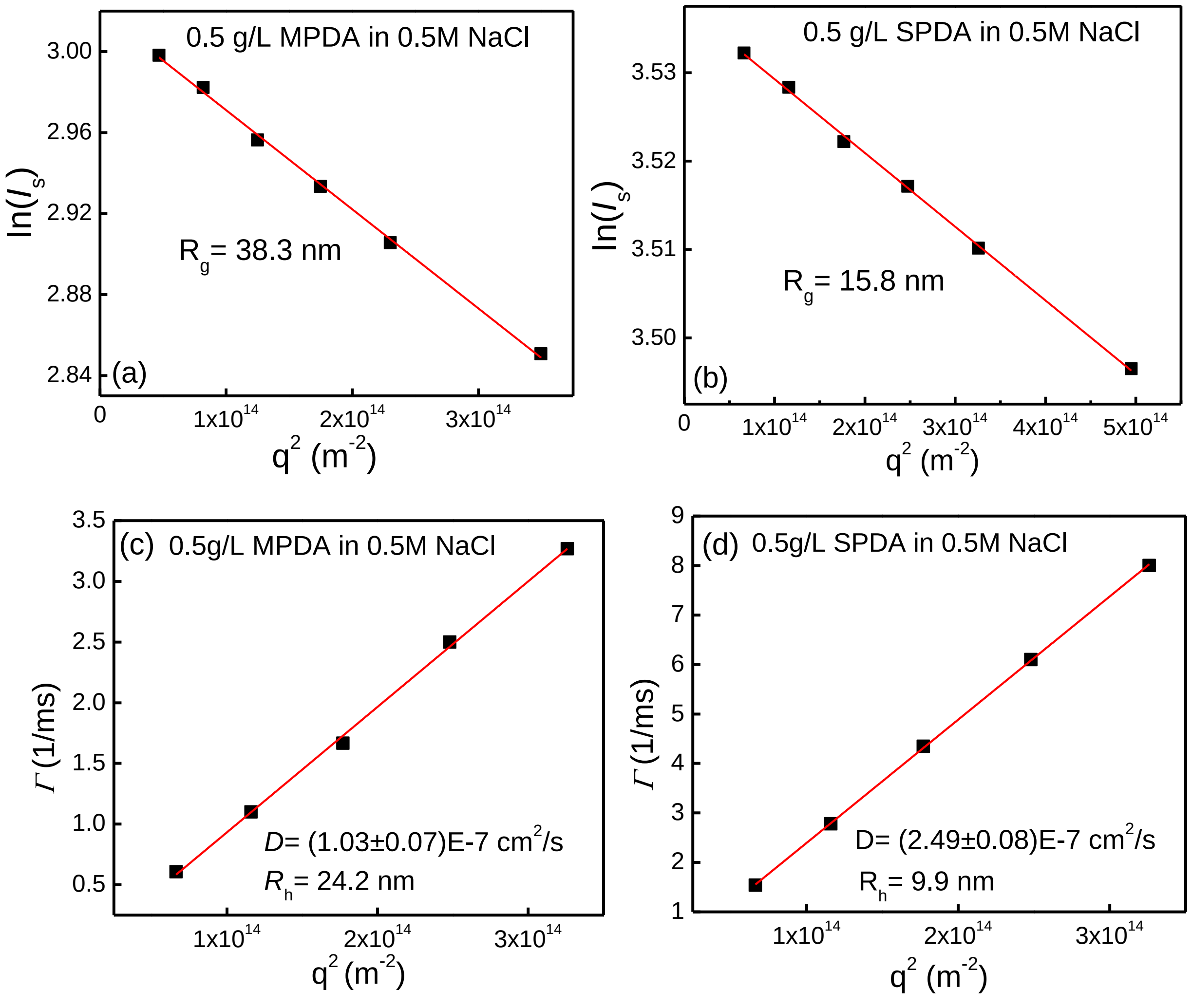

Supplementary Fig 1. Characterization of the guest chains of 0.5g/L MPDA and 0.5g/L SPDA in 0.5M NaCl solution. Guinier plot of $\ln(I_s)$-q^2 of (a) MPDA and (b) SPDA. q^2 dependence of the relaxation rate Γ to obtain the diffusion coefficient D of (c) MPDA and (d) SPDA.

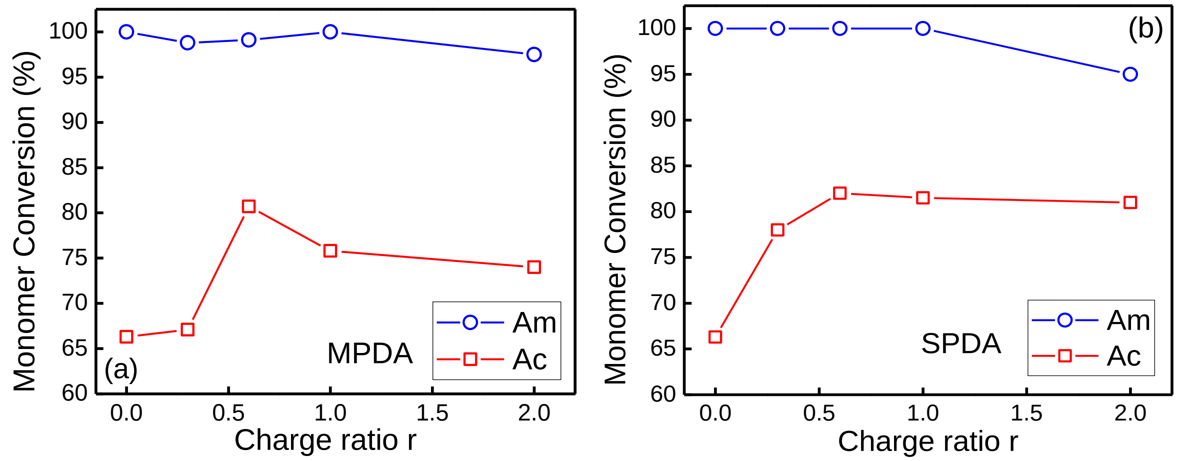

Supplementary Fig 2. NMR measurements for gel composites. Chemical conversion of Am monomers and Ac monomers of gel composites with (a) MPDA and (b) SPDA at different molar charge ratio r=0, 0.3, 0.6, 1, 2, respectively.

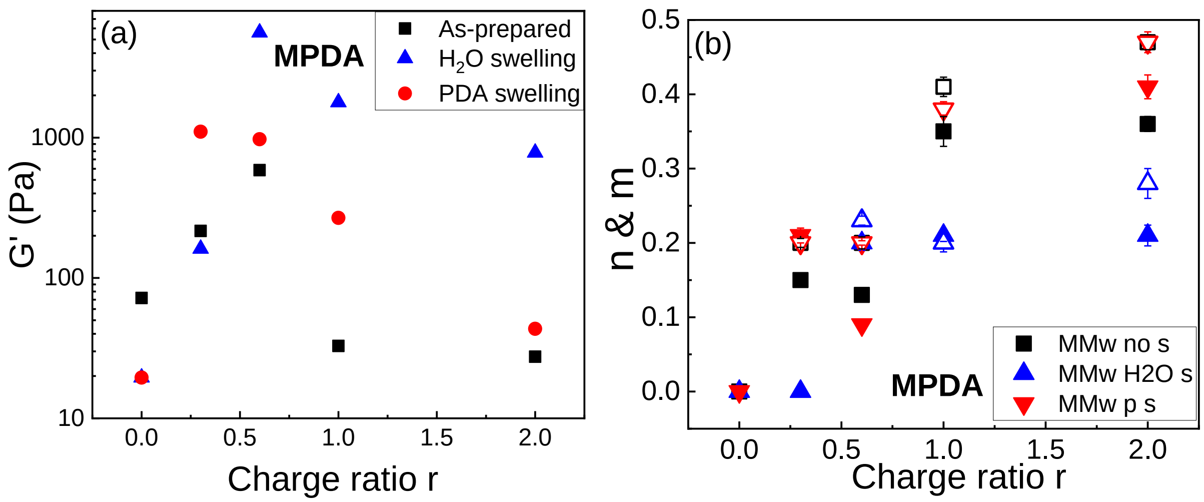

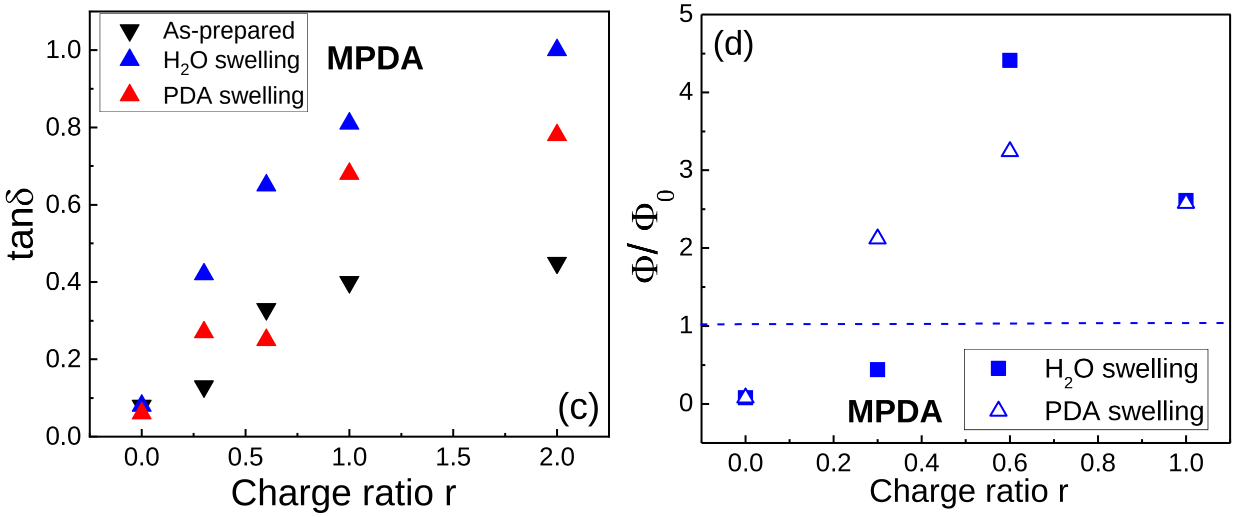

Supplementary Fig 3. Viscoelastic properties of the gel composites with MPDA. (a)-(c) Map of elastic modulus and viscoelastic properties in the PDA-gel matrix complexation. All the data were obtained from dynamic frequency sweeps at ω=1 rad/s. (a) Elastic modulus G', (b) Fitting values of n, m ($G' \sim \omega^n$, $G'' \sim \omega^m$), and (c) tanδ values (tanδ=G''/G') at different molar charge ratio r for gel composites with MPDA respectively. Three states were measured for each sample: as-prepared state, swelled by deionized water, and swelled by PDA solutions, whose polymer concentration is the same as that inside the gel composite. (d) Swelling ratio Φ/Φ_0 at different r for gel composites with MPDA swelled by water and PDA solutions. Each of the data points denotes the average of three individual replicates, and error bars are ± sd.
